# Longitudinal co-variation of radiographic chest-wall thickness and transcutaneous–arterial CO₂ discrepancy in congenital diaphragmatic hernia: a single-patient case study

**DOI:** 10.3389/fped.2026.1887546

**Published:** 2026-09-23

**Authors:** Tomonori Kurimoto, Eiji Hirakawa, Hiroshi Ohashi, Takuya Tokuhisa

**Affiliations:** 1 Department of Pediatric Cardiology, National Cerebral and Cardiovascular Center, Suita-shi, Osaka, Japan; 2 Department of Neonatology, Perinatal Medical Center, Kagoshima City Hospital, Kagoshima, Japan

**Keywords:** congenital diaphragmatic hernia, measurement discrepancy, neonates, radiographic chest-wall thickness, transcutaneous CO2

## Abstract

**Background:**

Transcutaneous carbon dioxide (tcPCO₂) monitoring enables continuous non-invasive assessment of ventilation in neonates, but local tissue conditions may contribute to disagreement between tcPCO₂ and arterial PaCO₂ measurements. We explored within-patient temporal co-variation among radiographic chest-wall thickness, sensor heating power, and the discrepancy between tcPCO₂ and arterial PaCO₂ in a critically ill neonate.

**Methods:**

We conducted an exploratory single-patient longitudinal case study over 28 days. Serial measurements of tcPCO₂, arterial PaCO₂, radiographic chest-wall thickness ratio (%skin), an unvalidated radiographic measure explored as a potential surrogate of tissue edema, sensor heating power, and daily fluid balance were evaluated. Both the signed difference (tcPCO₂−PaCO₂) and absolute difference |tcPCO₂−PaCO₂| were summarized descriptively. Temporal trajectories and within-patient associations were explored with attention to serial dependence and shared time trends.

**Results:**

Increasing radiographic chest-wall thickness coincided with greater tcPCO₂–PaCO₂ discrepancy and higher sensor heating power. The three variables showed broadly similar rise-and-fall temporal trajectories. Unadjusted within-patient correlations were strong (*r* = 0.766–0.813), and residual correlations persisted but were attenuated after removal of the quadratic Day/Day^2^ trend (residual *r* = 0.545–0.625). These residual correlations were interpreted descriptively and not as evidence of a time-independent relationship. PCA identified a dominant principal component explaining 85.9% of total variance, descriptively summarizing shared temporal covariance among radiographic chest-wall thickness, sensor heating power, and tcPCO₂–PaCO₂ discrepancy.

**Conclusion:**

In this single-patient longitudinal case study, radiographic chest-wall thickness, sensor heating power, and tcPCO₂–PaCO₂ discrepancy showed parallel temporal changes. These findings generate the hypothesis that changes in local tissue conditions associated with increased radiographic chest-wall thickness may contribute to tcPCO₂–PaCO₂ disagreement, but causal inference is not possible and validation in multi-patient studies is required.

## Introduction

Continuous monitoring of carbon dioxide is critical in neonatal intensive care. Transcutaneous partial pressure of carbon dioxide (tcPCO₂) provides a non-invasive method that reduces the need for frequent arterial blood gas sampling, thereby potentially limiting iatrogenic anemia (1–3). While tcPCO₂ is widely used during mechanical ventilation, therapeutic hypothermia, and neonatal transport, agreement between tcPCO₂ and arterial PaCO₂ may vary under certain clinical conditions (4, 5). Edema, often present in critically ill neonates, may increase local tissue thickness and alter microcirculatory conditions. These changes may alter gas diffusion dynamics and lead to discrepancies between tcPCO₂ and arterial CO₂ values (6). However, the relationship between tissue edema and disagreement between tcPCO₂ and arterial PaCO₂ measurements remains poorly characterized. We therefore conducted an exploratory single-patient longitudinal analysis to examine temporal associations among radiographic chest-wall thickness, sensor heating power, and tcPCO₂–PaCO₂ discrepancy.

## Methods

### Clinical and physiological course

We report a late-preterm neonate (gestational age 37 + 6 weeks, birth weight 2,209 g) with prenatally diagnosed congenital diaphragmatic hernia (CDH) associated with intrathoracic liver position. In accordance with standard practice for prenatally diagnosed cases with thoracic liver herniation, the infant was immediately intubated in the delivery room to avoid oronasal positive pressure ventilation and potential gastric distension. High-frequency oscillatory ventilation (HFOV) was initiated due to severe pulmonary hypoplasia and hypercapnia. A nasogastric tube was placed and connected to continuous suction for decompression of abdominal contents, thereby minimizing pulmonary compression. Umbilical arterial and venous catheters were inserted for hemodynamic monitoring and administration of fluids and medications. Hemodynamic support included isotonic fluids and inotropic agents to reduce right-to-left shunting.

Ventilation management followed a lung-protective strategy, with HFOV settings titrated to achieve optimal lung expansion (mean airway pressure 10–12 cm H₂O, frequency 8–12 Hz) and oxygenation targets (preductal SpO₂ 91%–95%) (7, 8). A permissive hypercapnia approach was applied, maintaining PaCO₂ between 45 and 65 mmHg and arterial pH 7.25–7.40 (9). Inhaled nitric oxide was initiated at 20 ppm when the oxygenation index (OI) reached 25 or higher (10, 11). Continuous sedation and analgesia were provided with vecuronium (0.05–0.1 mg/kg/h), fentanyl (2 µg/kg/h), and dexmedetomidine (0.4 µg/kg/h). Despite ongoing respiratory insufficiency, ECMO was not required since the OI remained stable without further progression.

Definitive surgical repair of the diaphragmatic defect was successfully performed on day 2 of life. We transitioned to assist-control ventilation on day 13, and the patient was successfully extubated on day 23, followed by Synchronized nasal intermittent positive pressure ventilation support.

### Measurements

tcPCO₂ monitoring was performed using a TCM5 FLEX transcutaneous monitor (Radiometer®, Copenhagen, Denmark). The transcutaneous sensor was calibrated according to the manufacturer's instructions before clinical use. Sensor temperature was maintained at 42 °C according to manufacturer recommendations (12, 13). tcPCO₂ values were obtained from the right lateral chest wall (4th rib region). Arterial blood gas measurements, including PaCO₂, were collected daily. Chest radiographs were used to calculate a radiographic chest-wall thickness ratio (%skin), an unvalidated radiographic measure explored as a potential surrogate of tissue edema.

Supine chest radiographs were obtained daily as part of routine clinical care during the study period. For the longitudinal analysis, one routine morning measurement set was used for each study day from Day 0 through Day 27. Arterial blood sampling was performed between 06:00 and 08:00, and the routine morning chest radiograph was obtained between 08:00 and 09:00. The interval between arterial blood sampling and radiograph acquisition was 1–3 h. Additional radiographs or blood gas measurements obtained at other times for clinical indications were not included in the longitudinal paired analysis. The radiographic chest-wall thickness ratio was defined as the ratio of (i) the distance from the right lateral skin edge to the lateral margin of the right fourth rib (numerator) to (ii) the distance from the skin to the center of the fourth vertebral body at the same horizontal level (denominator), multiplied by 100. To assess intra-rater reliability, radiographic chest-wall thickness measurements were repeated by the same investigator on a separate occasion. Intra-rater reliability was assessed using the intraclass correlation coefficient (ICC). The ratio was selected to partially normalize for differences in radiographic magnification; however, it has not been validated as a quantitative measure of neonatal tissue edema. Because the denominator geometrically includes the numerator (i.e., the skin-to-vertebral-center distance comprises the skin-to-rib distance plus the rib-to-vertebral-center distance), the numerator and denominator are not independent measurements. To clarify how changes in the underlying measured distances contributed to changes in the ratio, the numerator and denominator were also examined descriptively over time. Sensor heating power (mW) was recorded as an objective indicator of the heating demand required to maintain the preset sensor temperature during tcPCO₂ monitoring. Fluid intake, output, and daily fluid balance were recorded. Daily fluid balance was normalized to birth weight rather than current body weight, because postnatal weight gain partly reflected edema and fluid retention. This approach was used to avoid underestimation of fluid load during the edematous phase.

The signed tcPCO₂–PaCO₂ difference was defined as tcPCO₂−PaCO₂, with positive values indicating higher tcPCO₂ than PaCO₂ and negative values indicating lower tcPCO₂ than PaCO₂. The absolute difference was defined as |tcPCO₂−PaCO₂| and was used as a descriptive measure of the magnitude of disagreement. We use “signed difference” and “absolute difference” throughout to distinguish the direction of disagreement from its magnitude.

For each arterial blood gas sample, the tcPCO₂ value displayed at the time of arterial sampling was recorded. Only measurements obtained after the displayed tcPCO₂ value had remained stable for at least 10 min were included. Stability was assessed clinically from the monitor display and was defined as an apparent plateau without a sustained upward or downward trend for at least 10 min; no prespecified numerical fluctuation threshold was used. All PaCO₂ measurements were performed using the same blood gas analyzer (ABL90 FLEX PLUS blood gas analyzer [Radiometer®, Copenhagen, Denmark]).

The sensor membrane was changed every 7 days in accordance with the manufacturer's recommendations. The sensor was routinely repositioned every 6 h to a different site on the right lateral chest wall, and all measurements used for this analysis were obtained from this region. All paired observations meeting these criteria were included in the analysis.

### Statistical analysis

Statistical analyses were considered exploratory because all repeated observations were obtained from a single patient and therefore did not represent independent biological replicates. Continuous variables were first examined graphically as longitudinal time series, and paired observations were displayed using scatterplots.

Longitudinal changes in continuous variables were described using quadratic regression models with linear (Day) and quadratic (Day^2^) terms as predictors in ordinary least squares (OLS) regression. These models were used to characterize temporal trajectories rather than for population-level statistical inference. The signed and absolute transcutaneous–arterial CO₂ differences (tcPCO₂–PaCO₂) were summarized descriptively.

Associations among radiographic chest-wall thickness ratio (%skin), sensor heating power (used as an indicator of local tissue heating demand during tcPCO₂ stabilization), and the signed tcPCO₂–PaCO₂ difference were evaluated using Pearson correlation coefficients. Because serial observations within a single patient are not statistically independent, Pearson correlation coefficients were interpreted as descriptive measures of within-patient temporal co-variation, and conventional *p*-values were not used for inferential interpretation.

Because these variables exhibited pronounced temporal trajectories, serial dependence was examined using autocorrelation functions (ACFs) of the raw series and the residuals from the quadratic time-trend models. To describe residual co-variation after removal of the modeled quadratic relationship with postnatal age, each variable was regressed on Day and Day^2^, and Pearson correlations among the resulting residuals were calculated. These residual correlations were treated as descriptive exploratory measures and were not interpreted as evidence of an association independent of time or of unmeasured time-varying confounding.

Principal component analysis (PCA) was performed on standardized variables to explore common variance structures among radiographic chest-wall thickness ratio, sensor heating power, and signed tcPCO₂–PaCO₂ difference over time. PCA was used solely as an exploratory descriptive analysis of shared temporal covariance. Because all observations originated from a single longitudinal time series, the PCA findings were not interpreted as evidence of external reproducibility, statistical validation, or an independent biological mechanism.

Given the exploratory single-patient design, emphasis was placed on the magnitude and temporal pattern of observed associations rather than conventional hypothesis testing. No population-level statistical inference was intended.

### Ethics statement

This study was conducted in accordance with the ethical standards of Kagoshima City Hospital and the Declaration of Helsinki. Ethical approval was waived because this study involved a retrospective observational analysis of routine clinical monitoring data from a single patient. Written informed consent for publication of anonymized clinical data and accompanying figures was obtained from the patient's parents.

## Results

The longitudinal analysis included 28 daily morning measurement sets from Day 0 through Day 27, each comprising the routine morning chest radiograph and the corresponding morning tcPCO₂/PaCO₂ pair. Intra-rater reliability of the radiographic chest-wall thickness ratio was excellent (ICC = 0.94). Both the numerator (skin-to-rib distance) and denominator (skin-to-vertebral-center distance) varied over time. The rib-to-vertebral-center component of the denominator showed comparatively less temporal variation than the skin-to-rib distance. Because the denominator geometrically includes the numerator, the numerator and denominator were not interpreted as independent measurements. All three variables showed pronounced nonlinear temporal changes during the observation period, characterized by an initial increase followed by a subsequent decline (Figure 1). Descriptive quadratic models including Day and Day^2^ were consistent with these rise-and-fall trajectories (Table 1). Because all measurements represented serial observations from a single patient, these models were used to characterize temporal patterns rather than for population-level statistical inference.

**Figure 1:**
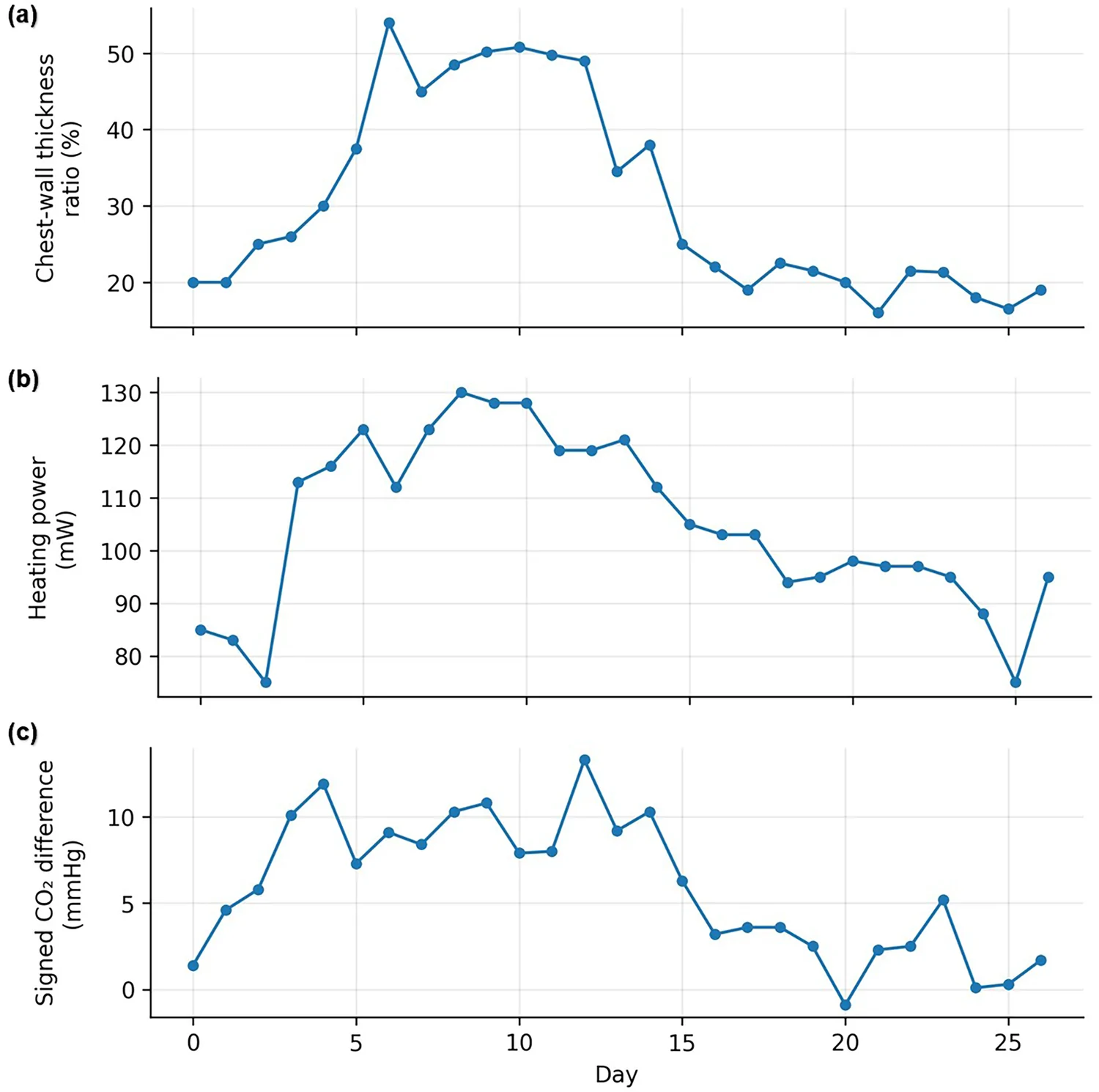
Longitudinal trajectories of radiographic chest-wall thickness ratio, sensor heating power, and signed tcPCO₂–PaCO₂ difference during the clinical course. **(a)** Serial changes in radiographic chest-wall thickness ratio (%skin), an unvalidated radiographic measure explored as a potential surrogate of tissue edema. **(b)** Longitudinal changes in sensor heating power required to maintain the preset sensor temperature. **(c)** Temporal changes in the signed tcPCO₂–PaCO₂ difference, defined as tcPCO₂ minus PaCO₂. Positive values indicate tcPCO₂ values higher than PaCO₂, whereas negative values indicate lower tcPCO₂ values. All measurements represent serial observations from a single patient.

**Table 1:** Descriptive quadratic models of longitudinal trajectories in radiographic chest-wall thickness ratio, sensor heating power, and signed tcPCO₂–PaCO₂ difference.

Variable	Term	Coefficient
Radiographic chest-wall thickness ratio (%skin)	Intercept	25.44
	Day	2.73
	Day^2^	−0.13
sensor heating power	Intercept	91.21
	Day	4.65
	Day^2^	−0.20
Signed tcPCO₂−PaCO₂ difference	Intercept	5.78
	Day	0.65
	Day^2^	−0.04

Quadratic models including Day and Day^2^ were used descriptively to characterize nonlinear temporal trajectories. The signed tcPCO₂–PaCO₂ difference was defined as tcPCO₂ minus PaCO₂. Because all observations were serial measurements from a single patient, the regression coefficients are presented solely as descriptive summaries of the observed temporal trajectories and should not be interpreted as population-level effect estimates or inferential statistics

The signed tcPCO₂–PaCO₂ difference was defined as tcPCO₂ minus PaCO₂. Unadjusted descriptive within-patient correlations were strong among radiographic chest-wall thickness ratio, sensor heating power, and signed tcPCO₂–PaCO₂ difference: *r* = 0.813 between radiographic chest-wall thickness ratio and sensor heating power, *r* = 0.784 between radiographic chest-wall thickness ratio and signed tcPCO₂–PaCO₂ difference, and *r* = 0.766 between sensor heating power and signed tcPCO₂–PaCO₂ difference. Raw paired observations are shown in Figure 2.

**Figure 2:**
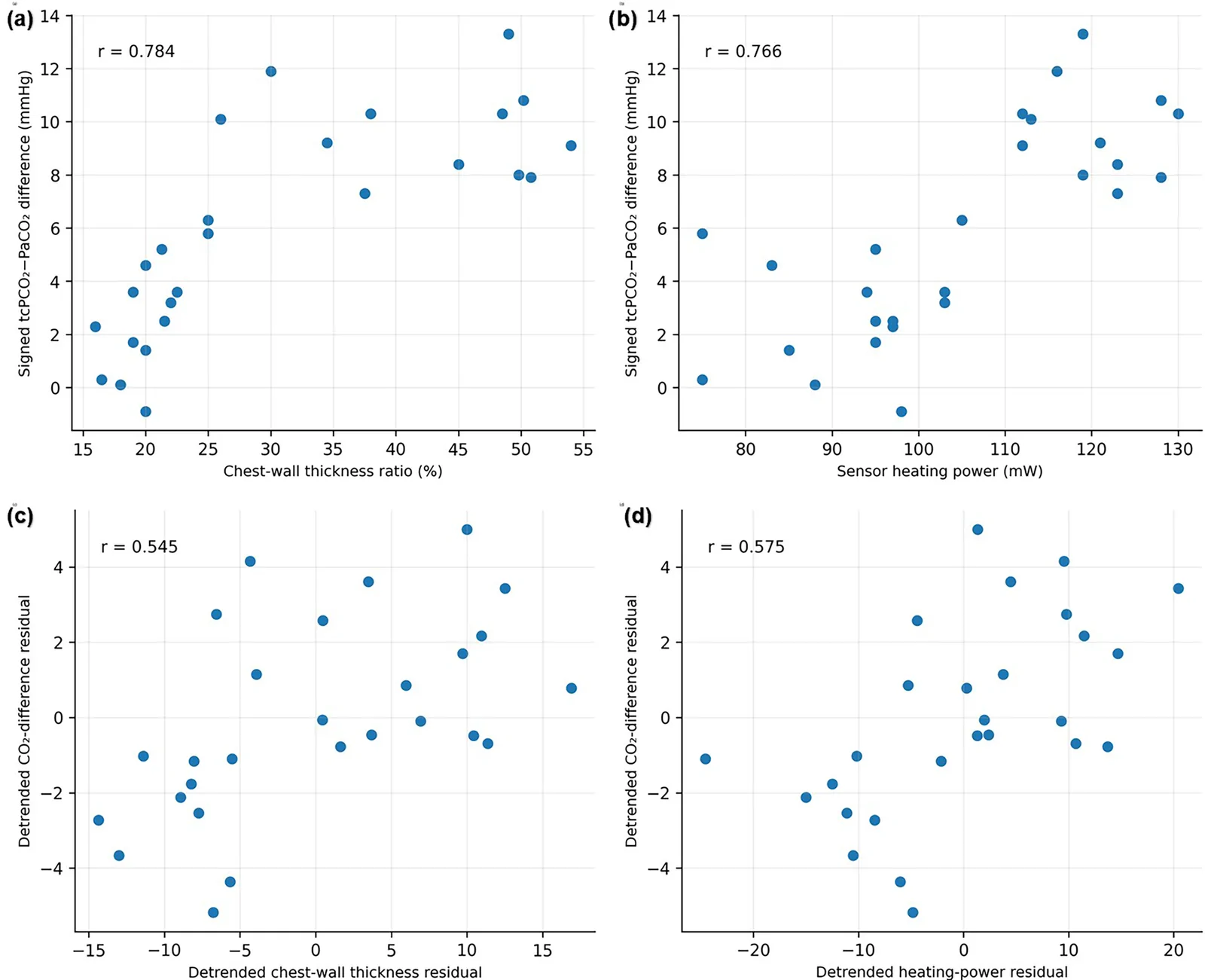
Raw and detrended within-patient associations between radiographic chest-wall thickness ratio, sensor heating power, and signed tcPCO₂−PaCO₂ difference. **(a)** Raw paired observations of radiographic chest-wall thickness ratio and signed tcPCO₂–PaCO₂ difference (*r* = 0.784). **(b)** Raw paired observations of sensor heating power and signed tcPCO₂–PaCO₂ difference (*r* = 0.766). **(c)** Association between residuals of radiographic chest-wall thickness ratio and signed tcPCO₂–PaCO₂ difference after removal of quadratic Day and Day^2^ trends (*r* = 0.545). **(d)** Association between residuals of sensor heating power and signed tcPCO₂–PaCO₂ difference after removal of quadratic Day and Day^2^ trends (*r* = 0.575). Each point represents a serial observation from the same patient. Correlation coefficients are presented as descriptive measures of within-patient temporal association and not as population-level inferential statistics.

Because the three variables exhibited similar nonlinear temporal trajectories, we additionally examined residual co-variation after removing the quadratic Day/Day^2^ trend. The correlations were attenuated but remained moderate after detrending: residual *r* = 0.625 between radiographic chest-wall thickness ratio and sensor heating power, *r* = 0.545 between radiographic chest-wall thickness ratio and signed tcPCO₂–PaCO₂ difference, and *r* = 0.575 between sensor heating power and signed tcPCO₂–PaCO₂ difference. Residual correlations therefore persisted after removal of the modeled quadratic time trend; however, these residual correlations cannot be interpreted as evidence of a time-independent relationship because residual serial dependence and unmeasured time-varying confounding remained. Autocorrelation analysis demonstrated substantial serial dependence in the raw time series (Supplementary Figure S1), with lag-1 autocorrelation coefficients of 0.86 for radiographic chest-wall thickness ratio, 0.76 for sensor heating power, and 0.72 for signed tcPCO₂–PaCO₂ difference. After removal of the quadratic temporal trend, the corresponding lag-1 autocorrelations decreased to 0.73, 0.44, and 0.37, respectively. Residual serial dependence remained, particularly for the radiographic chest-wall thickness ratio; therefore, conventional correlation *p*-values were not interpreted as evidence of population-level statistical significance. Only one paired measurement had a negative signed tcPCO₂–PaCO₂ difference (−0.9 mmHg). Accordingly, analysis of the absolute difference yielded similar descriptive findings; the correlation between radiographic chest-wall thickness ratio and absolute tcPCO₂–PaCO₂ difference was *r* = 0.791 before detrending and *r* = 0.550 after detrending.

Exploratory PCA identified a first component explaining 85.9% of total variance, with similar loadings across the three variables (Supplementary Table S1). This finding was interpreted as a descriptive summary of their shared temporal covariance within this patient rather than as independent evidence of a common biological mechanism or reproducibility across patients.

Positive fluid balance was prominent during the early clinical course and subsequently decreased (Figure 3). Fluid balance was not included in the primary association analyses and is therefore presented descriptively rather than interpreted as an independent contributor to tcPCO₂–PaCO₂ disagreement.

**Figure 3:**
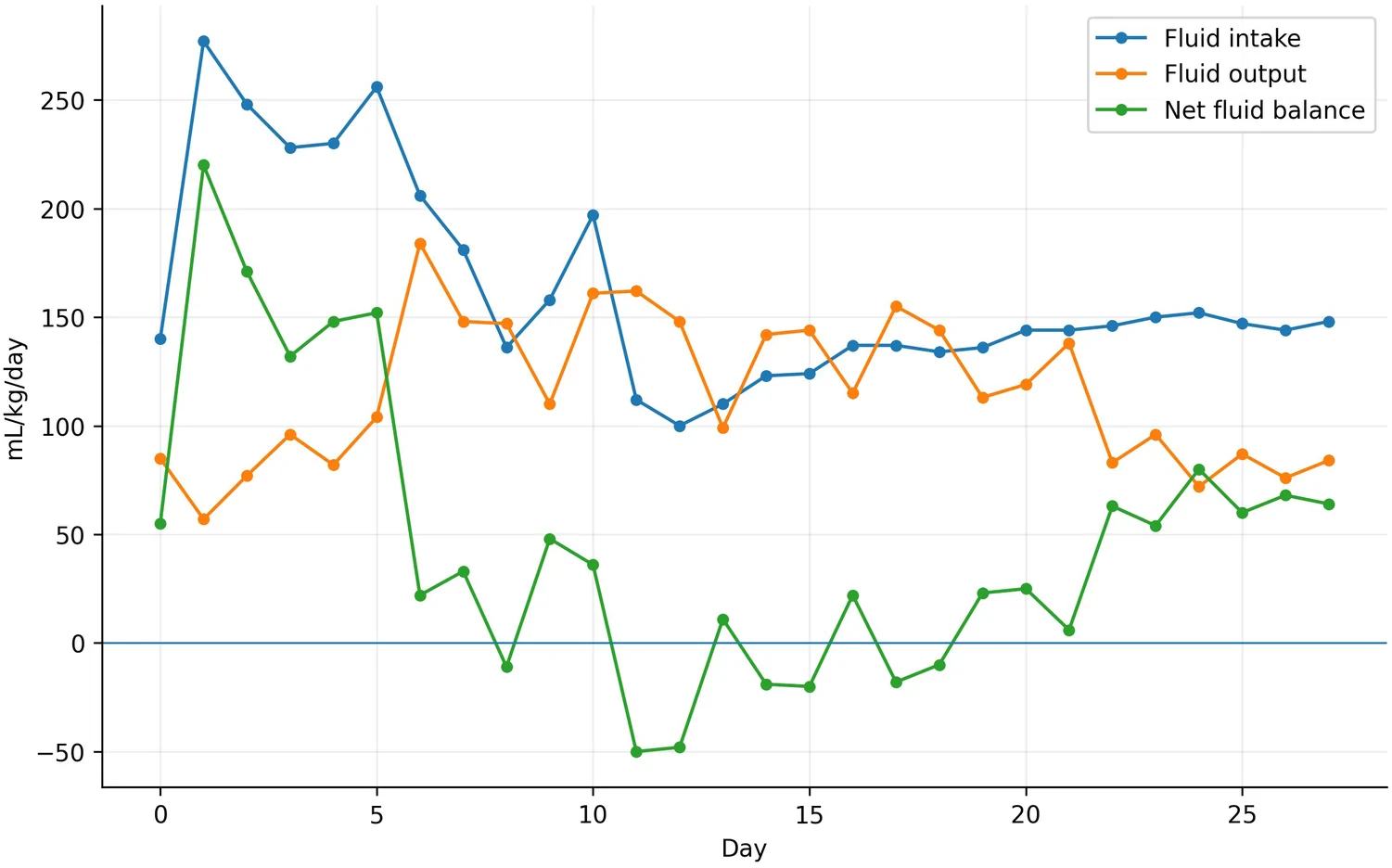
Longitudinal changes in daily fluid intake, output, and net fluid balance during the clinical course. Daily fluid intake, fluid output, and net fluid balance are expressed as mL/kg/day normalized to birth weight from Day 0 to Day 27. Fluid balance is presented descriptively to provide clinical context and was not included in the primary association analyses.

## Discussion

In this exploratory longitudinal case study of a late-preterm neonate with congenital diaphragmatic hernia, increases in radiographic chest-wall thickness ratio (%skin) coincided with widening discrepancies between tcPCO₂ and arterial PaCO₂. Using longitudinal and exploratory association analyses, we observed that radiographic chest-wall thickness, sensor heating power, and the tcPCO₂–PaCO₂ discrepancy followed broadly similar temporal trajectories. Positive fluid balance was also observed during the edematous phase but was not included in the primary association analyses and therefore should be interpreted descriptively. Residual correlations persisted after removal of the modeled quadratic time trend but were attenuated. Because residual serial dependence and unmeasured time-varying confounding remained, these residual correlations cannot be interpreted as evidence of a time-independent or independent relationship. These observations describe within-patient temporal co-variation among the measured variables but do not establish that edema independently contributed to tcPCO₂–PaCO₂ disagreement. Previous studies have highlighted several factors that may affect agreement between tcPCO₂ and arterial PaCO₂, including hypoperfusion, hypothermia, and probe-related technical issues (4–6).

In the present case, greater radiographic chest-wall thickness coincided with a larger tcPCO₂–PaCO₂ discrepancy and higher sensor heating power. One possible explanation is that edematous tissue altered local CO₂ diffusion, heat transfer, or microcirculatory conditions; however, none of these mechanisms was directly measured. Accordingly, these physiological explanations should be regarded as hypotheses generated by the observed temporal association rather than demonstrated mechanisms. Similarly, sensor heating power reflects the heating demand required to maintain the preset electrode temperature and should not be interpreted as a direct measure of local tissue perfusion. Exploratory PCA similarly summarized the shared temporal covariance among radiographic chest-wall thickness, sensor heating power, and tcPCO₂–PaCO₂ discrepancy, with PC1 accounting for approximately 86% of the observed variance. However, because all measurements were serial observations from a single patient and exhibited temporal trends, this finding should be interpreted descriptively and does not establish an independent effect of edema or the reproducibility of the observed relationship. From a clinical perspective, this case raises the possibility that increases in radiographic chest-wall thickness or associated changes in local tissue conditions may coincide with greater tcPCO₂–PaCO₂ disagreement. Increased sensor heating power may also provide contextual information about local measurement conditions; however, the present data do not establish it as a validated marker of impaired tcPCO₂ reliability. These single-patient observations are hypothesis-generating and do not establish that edema itself alters tcPCO₂ measurement performance. When marked edema or rapidly changing fluid status is present, disagreement between tcPCO₂ and PaCO₂ may warrant confirmation with arterial blood gas measurement before major ventilatory adjustments are made. However, the present single-patient observations are insufficient to define a clinically relevant threshold of edema or sensor heating power.

This analysis has several important limitations. First, all observations were repeated measurements from a single patient and therefore do not constitute independent biological replicates. Serial autocorrelation and shared temporal trends may inflate apparent associations among variables, and the findings should be interpreted as within-patient temporal associations rather than population-level effect estimates. Although exploratory detrending attenuated the observed correlations, residual serial dependence remained, particularly for radiographic chest-wall thickness; therefore, conventional correlation *p*-values cannot be interpreted as providing population-level statistical evidenc**e.** Moreover, residual correlations after quadratic detrending should not be interpreted as demonstrating a relationship independent of time, because detrending cannot account for unmeasured time-varying confounding. Second, the parallel evolution of radiographic chest-wall thickness, **s**ensor heating power, and tcPCO₂–PaCO₂ discrepancy occurred during substantial changes in illness severity and treatment, including ventilatory support, pulmonary hypertension management, vasoactive therapy, sedation, surgery, and changes in systemic and peripheral perfusion. These time-varying factors could not be adequately separated from the observed associations. Third, the radiographic chest-wall thickness ratio used as a surrogate of edema has not been validated for this purpose and may be influenced by patient rotation, radiographic magnification, arm position, respiratory phase, lung volume, chest-wall deformation, postoperative anatomical changes, and external devices. Although intra-rater reliability was excellent (ICC = 0.94), inter-rater reliability was not assessed. Fourth, sensor-site effects, repositioning, membrane changes, and sensor drift may have influenced tcPCO₂ measurements. Fifth, daily paired measurements provide only limited sampling of continuously changing physiological conditions. Sixth, CO₂ diffusion, local tissue perfusion, and heat transfer were not directly measured; therefore, the proposed mechanisms linking edema to tcPCO₂ discrepancy remain speculative. Finally, PCA of three variables from a single non-stationary time series provides a descriptive representation of shared covariance but cannot establish reproducibility, causality, or generalizability. Validation in a multi-patient cohort using standardized edema assessment and prospectively synchronized tcPCO₂–PaCO₂ measurements is required.

## Conclusion

In this single-patient longitudinal case study, radiographic chest-wall thickness, sensor heating power, and tcPCO₂–PaCO₂ discrepancy showed parallel temporal changes during the clinical course. Residual correlations persisted but were attenuated after removal of the modeled quadratic time trend; these residual correlations cannot be interpreted as evidence of a time-independent relationship. The observed temporal co-variation generates the hypothesis that changes in local tissue conditions may accompany tcPCO₂–PaCO₂ disagreement; however, radiographic chest-wall thickness is an unvalidated surrogate for edema, and shared time trends, serial dependence, unmeasured time-varying confounding, and concurrent changes in illness severity and treatment preclude causal or independent-effect inference. Multi-patient prospective studies with standardized edema assessment and synchronized tcPCO₂–PaCO₂ measurements are needed to determine whether this temporal co-variation is reproducible and clinically relevant.

## Data Availability

The datasets generated and/or analyzed during the current study are not publicly available due to patient privacy and institutional restrictions. Requests to access the datasets should be directed to the corresponding author.

## Supplementary material

SUPPLEMENTARY FIGURE S1 Autocorrelation of raw longitudinal series and residuals after removal of quadratic temporal trends Autocorrelation functions (ACFs) are shown for the raw longitudinal series of (a) radiographic chest-wall thickness ratio (%skin), (b) sensor heating power, and (c) signed tcPCO₂−PaCO₂ difference, and for the corresponding residuals after removal of quadratic temporal trends including Day and Day²: (d) radiographic chest-wall thickness ratio, (e) sensor heating power, and (f) signed tcPCO₂−PaCO₂ difference. The raw series demonstrated serial dependence, which was reduced after removal of the quadratic temporal trend, although residual autocorrelation remained, particularly for radiographic chest-wall thickness ratio. ACFs are presented descriptively because all observations were serial measurements from a single patient.
