# Supplementary material for: Longitudinal co-variation of radiographic chest-wall thickness and transcutaneous–arterial CO₂ discrepancy in congenital diaphragmatic hernia: a single-patient case study
Source: Front Pediatr. 2026 Sep 23;14:1887546. doi: 10.3389/fped.2026.1887546 (PMC13644392; doi:10.3389/fped.2026.1887546)
Supplement: Supplementary file 1 [file Table1.pdf]

Supplementary Table S1: Exploratory principal component analysis of the three longitudinal variables

	PC1	PC2	PC3
Radiographic chest-wall thickness ratio (%skin)	–0.583	–0.266	0.768
Sensor heating power	–0.578	–0.528	–0.622
Signed $tcPCO_2-PaCO_2$ difference	–0.571	0.806	–0.154

Principal component analysis (PCA) was performed to descriptively summarize the shared covariance structure among radiographic chest-wall thickness ratio (%skin), sensor heating power, and signed $tcPCO_2-PaCO_2$ difference. PC1 accounted for 85.9% of the total variance. Because all observations originated from a single longitudinal time series, PCA should not be interpreted as evidence of a common causal mechanism, statistical validation, or external reproducibility.

Supplementary Figure S1: Autocorrelation of raw longitudinal series and residuals after removal of quadratic temporal trends

Autocorrelation functions (ACFs) are shown for the raw longitudinal series of (a) radiographic chest-wall thickness ratio (%skin), (b) sensor heating power, and (c) signed $tcPCO_2-PaCO_2$ difference, and for the corresponding residuals after removal of quadratic temporal trends including Day and Day^2: (d) radiographic chest-wall thickness ratio, (e) sensor heating power, and (f) signed $tcPCO_2-PaCO_2$ difference. The raw series demonstrated serial dependence, which was reduced after removal of the quadratic temporal trend, although residual autocorrelation remained, particularly for radiographic chest-wall thickness ratio. ACFs are presented descriptively because all observations were serial measurements from a single patient.
